# Modern Sociology

**Published:** 1899-09-23

**Authors:** 


### Modern Sociology.

### THE ETHICS OF SUICIDE.—IV.

By Francis Scougall.

Many strange anomalies rise before me when I turn to the question of suicide as it affects the upper classes. Certain motives which tend to self-destruction among uneducated persons do undoubtedly often influence their superiors also, such as a condition of pecuniary ruin, intensified in its desperate hopelessness by the fact that it has been self-wrought, or a determination not to bear a long course of physical pain certain to result from a deadly malady; and many other forms of a sickening of life may thus be shared by those of all ranks, and especially through the same disbelief of any survival after death. The strange feature of this similitude of motive is, its power to influence the minds of those who can take a much wider range of all the issues of life, and can estimate higher principles of human action than can be apparent to their humbler brethren. On this ground there are at least two aspects of suicide which I should expect to act as stringent deterrents in men of culture and intellect, and these are its shameful cowardice and its unspeakable folly. The man—be he atheist or not—knows absolutely nothing of the hidden nature of life, of its possibilities and its meanings; but he knows that it is his possession for a few years only, that even eighty years of existence fades from the old man as a passing dream. While it is his possession he can make it noble; he can offer it as a royal gift of help and utility to his fellow creatures; he can render it eloquent of endurance, of unselfishness, of principles that shall raise all around him to a higher standpoint than they could otherwise have known. But to gratify his own low grovelling dread of pain, physical or mental, he flings it all with its possible promise into the dust of death, and so compasses his own pitiable humiliation. Can there be a more miserable spectacle than that of a man who has fallen from his integrity, and brought dishonour and disaster on himself and all connected with him; when, instead of having the manliness to admit his fault and face its penalty, he flings himself blindfold into unknown conditions, and leaves dire disgrace and pain to be the heritage of those he should have striven to shield? Dishonest and falsified accounts, about to be discovered in a man who has been trusted with the money of others, is well known to be one of the most fruitful sources of suicide. I admit, of course, that sometimes there are nobler motives for this fatal act—influencing even those who for their own sakes might desire to live—when their financial position is such that their family or friends would seem likely to be greatly benefited by their removal; but, generally speaking, this impression proves to be a fallacy, or, even if more or less true, it does not lessen the bitter grief probably felt by those for whom the sacrifice is made; nor can it in any way diminish the force of that truth which attaches to all cases of self-destruction—that it is an act of inconceivable folly. Even materialists, who refuse to recognise the existence of anything of which they have not personal knowledge, must admit the fact that a mysterious life has come to them by no agency of their own, and that so long as it endures, reigning with unknown powers in brain and mind and faculties, it has the potentiality of events, of marvels, of conditions affecting the individual, for which he would gladly wait if he could foresee their possible advent. Yet all this he flings away for the mere animal gratification of escaping at once a sense of mental or physical pain. Surely I have good reason to say that under all circumstances the demoralising act of suicide is also emphatically one of incredible folly—and it sins against all the nobler instincts of the human nature. I come now to the point towards which I have been aiming from the first. The criminality and folly of self-destruction, must after all be patent to all honest thinkers, and it is certainly a very strange and remarkable fact that no serious, strongly-organised protest against it, seems ever to have been made by the great majority of those who hold that their vocation is to teach and influence their fellow men. The innumerable churches of this land teem week after week with the utterances of earnest men, who are for ever striving to raise their fellow creatures to a higher level in all that is pure and of good report. Yet who can say that they have ever heard a sermon directed wholly and solely against the crime of self-murder? In our parks and on many public platform, the people may hear continually the voice of energetic leaders of thought who try to influence their hearers on politics, socialistic principles, creeds, and no creeds many and various; yet, amongst them all, who can say that he has heard a lecture delivered of which the theme was an unadulterated denunciation of suicide? So it is with the unbounded stores of the literary market; incidental treatises dealing with the subject may have appeared, and in the pages of fiction it is, of course, made use of continually as one of the most convenient and dramatic modes of disposing of an inconvenient character, but all this means nothing as a deterring influence. Now why should there not be once for all an organised, almost universal effort, to fight the hideous sacrifice of human life which goes under the name of self-destruction? The calm indifferent announcement in every daily paper of another human being flung away from all the uses of this world, into self-sought darkness and oblivion does not, as a rule, win a moment's attention from those unconnected with the actual victim to senseless folly and selfishness. Is it well that we should acquiesce with such complacent apathy in a fruitless holocaust, which drains our own country, at least, of a far greater proportion of men and women who might have been useful in their generation than is at all generally realised? There is no lack of altruism in the present day. Let, therefore, all the more prominent men of intellect and sterling sense, who desire to work for the good of others, take counsel together, and bring to practical working power a scheme for a well organised crusade against suicide. If at one and the same time men of eloquence and power would stand up in public places, where crowds could be drawn round them, to denounce this crying evil—to point out its guilt, its madness, its humiliating squandering of God's most imperial gift, I believe that an effect might be produced which would be of incredible value, and would astonish all interested on the subject.

